# Prosthetic Joint Infections in Trapeziometacarpal Arthroplasty: A Comprehensive Systematic Review

**DOI:** 10.3390/jpm16010035

**Published:** 2026-01-05

**Authors:** Guido Bocchino, Silvia Pietramala, Stella La Rocca, Giulia Di Pietro, Alessandro El Motassime, Giacomo Capece, Domenico De Mauro, Camillo Fulchignoni, Giulio Maccauro, Raffaele Vitiello

**Affiliations:** 1 Department of Orthopedics, Ageing and Rheumatological Sciences, Fondazione Policlinico Universitario A. Gemelli IRCCS, Largo Agostino Gemelli 8, 00168 Rome, Italy; guido.bocchino@hotmail.it (G.B.); silvia.pietramala01@icatt.it (S.P.); stella.larocca01@icatt.it (S.L.R.); giulia.dipietro05@icatt.it (G.D.P.); alessandro.elmotassime01@icatt.it (A.E.M.); domenico.demauro01@icatt.it (D.D.M.); camillo.fulchignoni@unicatt.it (C.F.); giulio.maccauro@unicatt.it (G.M.); raffaele.vitiello@unicatt.it (R.V.); 2 Orthopedics and Trauma Surgery, Catholic University of the Sacred Heart, 00168 Rome, Italy; 3 Pellegrini Hospital-U.O.C. Orthopaedics and Traumatology, 80134 Naples, Italy

**Keywords:** trapeziometacarpal arthroplasty, prosthetic joint infections, rhizarthrosis, infection management

## Abstract

**Background:** Osteoarthritisof the first trapeziometacarpal (TMC) joint (rhizarthrosis) is a degenerative condition causing pain, reduced mobility, and functional limitations, particularly in older adults and postmenopausal women. Though conservative treatments offer symptomatic relief, advanced cases often require trapeziectomy or total joint replacement. The choice of prosthesis is tailored to patient-specific factors such as age, functional demands, and comorbidities. Despite the benefits of TMC joint replacements, prosthetic infections remain underexplored. **Materials and Methods:** This systematic review (covering 2000–2024) adhered to PRISMA guidelines, searching Medline, Cochrane, and Google Scholar for randomized controlled trials and case series. Data on demographics, prosthesis types, infection rates, and management strategies were extracted and analyzed. **Results:** Among 4165 TMC joint procedures reported in 63 studies, 15 cases (0.36%) involved superficial or deep infections, with *Staphylococcus aureus* identified in two instances. Management ranged from antibiotic therapy and debridement to prosthesis removal with or without reimplantation. **Conclusions:** Variability in diagnostic criteria and reporting limited uniform conclusions. Although infections are infrequent, they pose significant management challenges due to inconsistent diagnostic criteria and treatments. Early identification and tailored interventions remain critical. This review underscores the need for standardized protocols and highlights gaps in current research. Future studies should focus on multicenter trials and robust methodologies to improve outcomes and advance infection management in TMC prosthesis surgery.

## 1. Introduction

**Figure 1:**
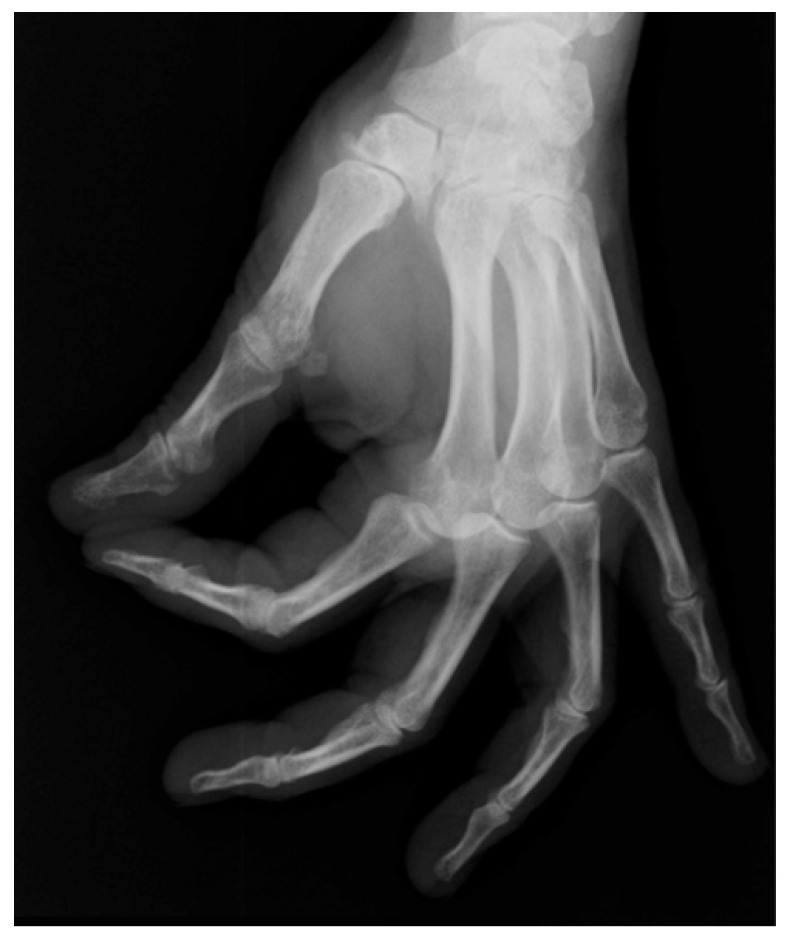
Radiographic image showing rhizarthrosis on a hand X-ray.

Osteoarthritis of the first trapeziometacarpal (TMC) joint, or rhizarthrosis, is a common condition, with an age-adjusted prevalence of 7% in men and 15% in women [1] (Figure 1). This condition can lead to pain, deformity, reduced range of motion, joint instability, and weakness, resulting in substantial functional limitations, particularly in postmenopausal women and older individuals [2]. The primary goals of treatment are to reduce pain, improve thumb mobility, enhance joint stability, and restore hand functionality [3]. Given the heterogeneity of patient characteristics, disease severity, functional demands, and comorbidities, the management of TMC osteoarthritis increasingly requires a personalized and precision medicine approach tailored to individual patient profiles.

Non-surgical management options include activity modification, oral analgesics, splinting, physical therapy, and corticosteroid injections [4]. Surgical intervention becomes necessary when conservative measures fail to adequately control symptoms. Surgical options include extension osteotomy, TMC arthroscopy with debridement, partial or complete trapeziectomy (alone or combined with ligament reconstruction and tendon interposition (LRTI) or suspensionplasty), arthrodesis, or joint replacement [2].

Trapeziectomy has been a standard surgical treatment for TMC osteoarthritis for over 70 years and is generally effective in relieving pain and restoring thumb mobility [4]. However, thumb shortening remains a concern, as it may impair pinch strength and result in impingement between the metacarpal base and the scaphoid. The addition of ligament reconstruction and tendon interposition was developed to address these challenges. Moreover, post-operative care requires a rigid cast for 3 to 4 weeks with an overall functional recovery time of 3 months. These limitations have highlighted the importance of selecting surgical strategies based on patient-specific functional needs, expectations, and risk factors, in line with principles of precision surgery.

**Figure 2:**
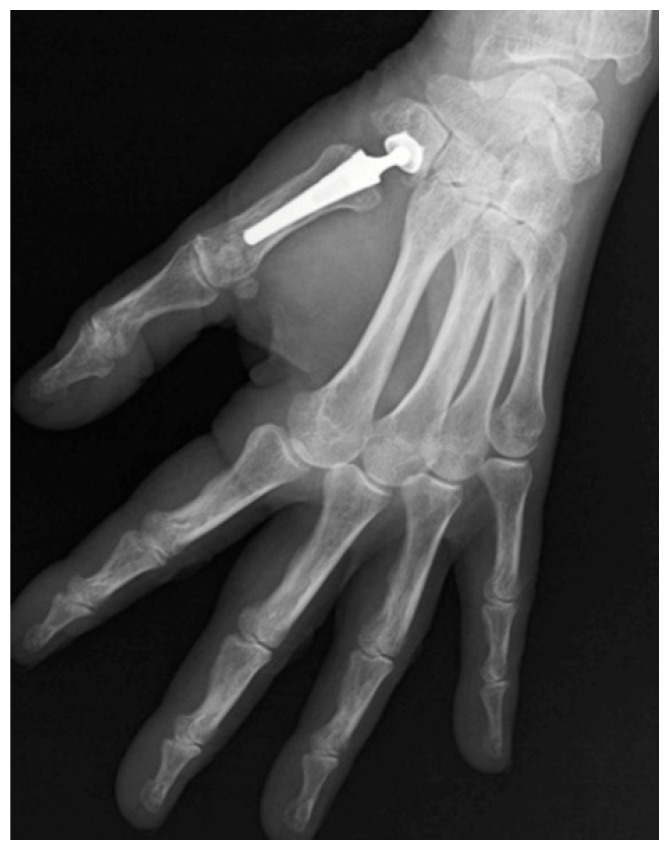
Radiographic image showing trapeziometacarpal joint prosthetic replacement.

On the other hand, most recent solutions, such as TMC arthroplasties, aim to restore thumb length while maintaining functional mobility, stability, and grip strength [5] (Figure 2). The initial generation of implants primarily utilized silicone spacers [6]. Subsequently, a total joint replacement that converted the TMC saddle joint into a ball-and-socket joint was introduced by integrating a cup in the trapezium and a cemented stem in the first metacarpal [7]. In recent years, attention has shifted towards cementless ball-and-socket implants with metal-on-polyethylene articulations, which aim to improve functionality and longevity while addressing the limitations of earlier designs. These technological advancements have expanded the range of implant options, enabling surgeons to better match implant characteristics to individual anatomical and functional requirements.

Modern cementless TMC implants use metal-on-polyethylene articulations with grid-blasted titanium or hydroxyapatite-coated cobalt-chrome components. These implants are available with various cup designs (e.g., hemispheric, conical, or screw) and stem configurations, as well as options for dual-mobility articulations.

Historically, total TMC joint replacements were considered inferior to trapeziectomy due to high revision rates and limited long-term implant durability. However, more recent non-randomized studies with follow-ups exceeding 12 months have demonstrated superior pinch and grip strength and faster recovery with joint replacements compared to trapeziectomy [8,9]. These findings have contributed to an increased preference for TMC joint replacements, challenging earlier perceptions of their limited benefits. Nonetheless, complications such as prosthetic joint infection, dislocation, loosening, and wear remain potential concerns [5]. Among these, prosthetic joint infection represents a particularly critical complication, as its risk and management may vary significantly according to patient-specific factors, surgical technique, and implant characteristics.

Research on complications related to TMC joint replacement surgery is sparse, with most studies focusing on single-surgeon experiences, older implant designs, and small patient cohorts. The primary aim of this review is to systematically summarize the available evidence on prosthetic infections following thumb CMC joint replacement, including their incidence, clinical presentation, treatment strategies, and outcomes. By synthesizing the existing evidence, this review seeks to support a personalized and precision medicine approach to risk stratification, prevention, and management of prosthetic joint infections in TMC arthroplasty. A secondary objective is to identify gaps in the current literature and provide evidence-based recommendations to guide clinical decision-making and future research in this underexplored area.

## 2. Materials and Methods

The review adhered to the PRISMA (Preferred Reporting Items for Systematic Reviews and Meta-Analyses) guidelines [10], ensuring a comprehensive and systematic approach to data retrieval and synthesis. The methodological approach was designed to capture patient-specific, surgical, and implant-related variables potentially relevant to personalized and precision medicine strategies in the prevention and management of prosthetic joint infections.

### 2.1. Search Strategy

The analysis was conducted using the keywords ‘trapeziometacarpal’, ‘rhizarthrosis’, ‘thumb arthritis’, ‘CMC’, ‘TM’, ‘TMC’, ‘thumb’ AND ‘prosthesis’, ‘Touch’, ‘replacement’, ‘Maia’, ‘Isis’. Databases searched included Medline (PubMED), Cochrane and Google Scholar up to 30 September 2024. Articles published in English, Spanish, French, Portuguese and Italian in peer-reviewed journals were considered. Excluded were biomechanical reports, animal studies, cadaver studies, in vitro research, case reports, case series with fewer than 10 cases, literature reviews, technical notes, letters to editors and instructional materials. Four authors (G.B, G.C, G.D.P, C.F.) independently reviewed abstracts and full texts were obtained if abstracts were inconclusive. All differences between the reviewers were discussed and if disagreement remained the senior author (R.V.) was consulted. Reference lists of selected articles were manually checked. All the selected studies were retrospectively analyzed by an author (G.B.) who then extracted and entered the data in an Excel worksheet. Lastly, the data sheet was reviewed by two authors (G.C, D.D.M.) who agreed on the extracted data.

The literature references of identified papers were also searched to find further relevant articles. All journals were considered.

### 2.2. Inclusion and Exclusion Criteria

The eligibility criteria for inclusion in this review were established to ensure the selection of high-quality studies. Included studies were randomized controlled trials (RCTs), clinical trials. Case reports and case series with fewer than 10 patients were also included if they reported prosthetic joint infections (PJI), due to the rarity and clinical relevance of these events. Excluded were systematic reviews, meta-analyses, in vitro studies, animal studies, cadaver studies, reports on other inflammatory conditions (e.g., tendinitis), and studies published in languages other than English, Spanish, French, Portuguese, or Italian. Studies reporting confirmed or suspected PJI were included. PJI was defined according to each study’s criteria, including microbiological evidence, clinical signs of infection, or requirement for surgical intervention (Table 1).

Three reviewers (G.C, S.L.R, C.F.) independently assessed the full texts of selected articles to determine eligibility and extracted relevant data. In cases of disagreement, the senior author (R.V.) made the final decision. Additionally, the risk of bias was evaluated for each study, with any disagreements resolved through consultation with the supervisor.

### 2.3. Data Extraction and Analysis

Detailed information was systematically extracted from each selected study. The selected studies covered a range of variables including demographic data, type of fracture, surgical methods, and outcomes related to return to sports. Statistical analysis was performed using SPSS 18.0 for Windows (SPSS Inc., Chicago, IL, USA). Descriptive statistics were used to summarize the findings across all the included studies.

## 3. Results

### 3.1. Search and Literature Selection

**Figure 3:**
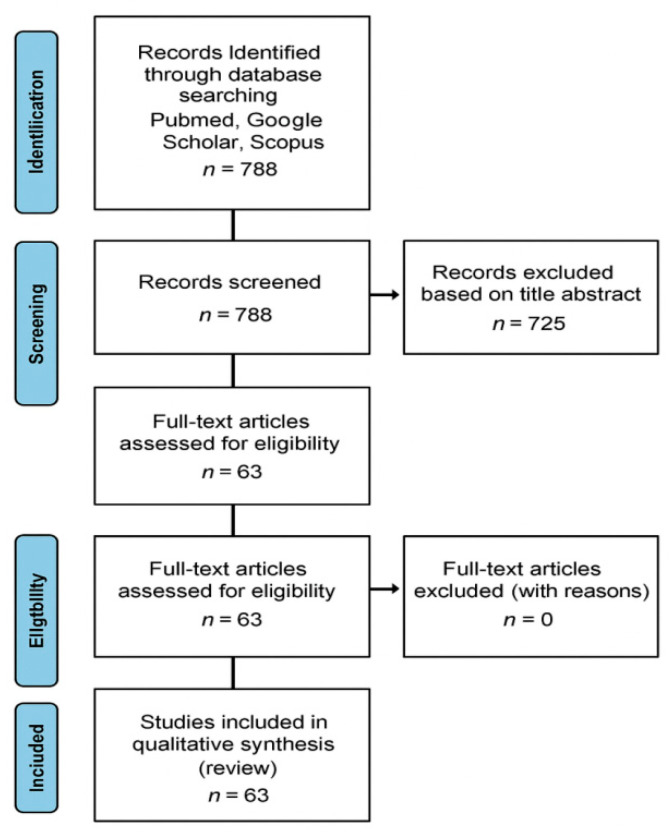
PRISMA Flow-chart.

The data analyzed come from scientific studies published between 2000 and 2024. An initial literature search identified 788 papers for potential evaluation. Out of these, 725 were discarded after reviewing their titles and abstracts, as they did not meet the inclusion criteria. In the end, 63 papers were included in the review (Figure 3). The checklist for this study can be found in the Supplementary Materials (Table S1).

### 3.2. Study Characteristics and Demographics

This systematic review includes data from 63 studies, including a total of 3573 patients who underwent surgical treatment for rhizarthrosis using a trapeziometacarpal joint prosthesis. Among these studies, 37 were retrospective, 19 were prospective and 5 were case reports. The mean age of participants was consistent across the studies, with an average age of 61.2 years. Regarding gender distribution, 569 patients were male (16%) and 2939 were female (82%), although 4 articles did not provide information on gender distribution [Table 2]. Additionally, 20 articles reported the occupational background of the patients, showing a high prevalence of implants among individuals who had engaged in manual work during their lives. The wide variability in patient age, sex distribution, occupational background, and implant selection highlights the heterogeneity of the treated population, supporting the relevance of patient-specific factors in the assessment of prosthetic joint infection risk.

### 3.3. Type of Prosthesis

The Maia prosthesis emerged as the most used implant, being reported in 10 out of 63 studies included in this review. It was followed, in descending order of frequency, by the Elektra (9/63), Arpe (9/63), Touch (7/63), Ivory (6/63), Moovis (6/63), Isis (3/63), Moje Acamo (3/63), SR TMC (3/63), Motec (2/63), Roseland (2/63), Guepar (2/63), Swanson (2/63), De la Caffiniére (2/63), Rubis II (2/63), and BioPro Modular implants (1/63).

### 3.4. Infection Rate

Out of 4165 procedures, 15 were complicated by infections. Among these, 6 cases involved superficial infections, while deep infections were reported in 8 cases. One case was classified as suspected deep infection due to the presence of purulent material in the joint and along the stem, although no pathogens were isolated. The specific pathogen was identified in only 2 cases, both involving *Staphylococcus aureus*; in the remaining cases, microbiological confirmation was not available, likely due to superficial infections, lack of synovial sampling, or incomplete reporting by the original authors. Only two superficial infections were successfully treated with oral antibiotics, while the treatment approach for the remaining cases was not specified. Surgical intervention was performed in 9 cases, including 3 trapeziectomies, 1 debridement with placement of a bone cement spacer and arthrodesis after 6 months, and 1 prosthesis explantation with conversion to resection arthroplasty. In one additional case, the prosthesis was removed, but the authors provided no further details regarding the procedure [Table 3]. In 5 cases the prosthesis involved was Maia. For most patients, detailed information regarding the time from surgery to onset of infection, the type of surgery performed, and the antibiotic treatment used was not available, limiting the ability to classify infections as early or late. Some of the reported infections were superficial and may not meet strict PJI criteria, highlighting the limited epidemiological information available.

## 4. Discussion

Over the years, TMC prostheses have become a reliable and widely appreciated solution for managing trapezio-metacarpal joint arthritis, offering significant benefits in pain relief, functionality, and quality of life [69]. The first TMC prosthesis was implanted in 1970 [47] and despite it being a long time, the global follow-up remains poor compared with hip and knee implants. While mechanical implant-related complications are more predictable [13], infection rate is something that has not been examined as, so far, no review addressed the topic. In this context, prosthetic joint infection represents a paradigmatic complication in which patient-specific factors, surgical variables, and implant-related characteristics interact, making a personalized and precision medicine approach particularly relevant. With an average implant survival rate of 90–94% after 10 years [17,48], surgeons must be able to detect and efficiently treat infections. Infection is a relatively uncommon complication in elective hand surgery, with an overall incidence of 1.9% [70]. Authors usually refer to infectious complications using the terms superficial SSI (surgical site infection) and deep infection. A first bias in determining the actual infection rate of TMC prosthesis is the appropriateness in classifying the type of infection. This lack of standardized definitions hampers risk stratification and limits the possibility of tailoring diagnostic and therapeutic strategies to individual patients.

According to Centers for Disease Control and Prevention (CDC) [71] a superficial SSI is defined as an infectious process that occurs within 30 days after the operation and that involves only skin or subcutaneous tissue of the incision. The local factors which identify an SSI are: (1) localized swelling, (2) redness, (3) heat while the presence of a stitch abscess with minimal inflammation and discharge confined to the points of suture penetration or SSI that extend into the fascial and muscle layers mustn’t be related to an SSI. The presence of discharge, especially around an exposed K-wire, is a common finding in hand surgery which can be easily mistaken for a sign of infection.

Accurate clinical interpretation is therefore essential to avoid overtreatment or undertreatment, particularly in frail or comorbid patients.

On the other hand, a deep infection occurs within 30 days if no implant is left in place or within 1 year in cases of implants in place. The main sign of deep infection is the presence of purulent drainage from the deep incision or a deep incision which spontaneously dehisces in patients who report fever, localized pain or tenderness. Another element which defines a deep infection is the presence of an abscess or other evidence of infection involving the deep incision. A limited number of studies in the literature specifically addresses infections in TMC. This scarcity of data highlights the pertinence of this review, which aims to provide a comprehensive analysis of infection rates, potential risk factors and recommended management strategies in TMC.

This scarcity of data highlights the importance of synthesizing available evidence to support individualized clinical decision-making in this highly heterogeneous patient population.

Our research found only 12 [6,8,11,12,16,20,35,37,41,46,59,67] articles addressing infections in TMC prosthesis with a total of 15 cases reported. In most cases [8,11,12,20,35,37,46,67] (9/6) a deep infection was described, and this could lead to a lack of data due to the absence of a report from surgeons in case of superficial and self-resolving infections. The overall incidence reported in our study is 0.36% across 4165 procedures which is low but not negligible, considering an infection rate of 0.3–0.5% in bigger joints such as hip and knee [72]. Due to lack of data regarding the onset of symptoms, it was not possible to estimate the rate of early and late infection which could be helpful in stratifying the causes [47,69,73]. This reduced incidence can be attributed to several factors, including the shorter operative time, reduced surgical exposure, and minimal instrumentation involved in TMP procedures [74]. Moreover, as pointed out by Kistler et al., much of the literature on infection rates in hand surgery is based on reoperation rates while ignoring infectious complications treated simply with outpatients antibiotics [75]. Physicians address prosthetic infections using Parvizi infection criteria, which might seem inappropriate [76].Parvizi infection criteria [76] lack usefulness in this field as synovial fluid aspiration is not a common practice also due to the limited articular space as well as histological analysis of the periprosthetic tissue. The limited applicability of existing diagnostic frameworks further supports the need for tailored criteria adapted to small-joint arthroplasty and individual patient characteristics. In our series, the responsible bacteria were identified in only 2 cases [6,12] (both *Staphylococcus aureus*) through microbiological samples obtained during revision surgery. CRP and ESR could be easily obtained in most cases along with WBC counts to assess infection, but no author specified whether they used them. Therapeutic strategies are heterogeneous and insufficiently detailed. Implant removal is implemented in some cases [6,33,37,45,55] but no consensus exists.

Treatment decisions should therefore be individualized, integrating infection chronicity, host factors, implant stability, microbiological data, and patient functional demands within a precision medicine framework. Medical management must be carried out in conjunction with an infectious diseases specialist, taking into account the patient’s condition, duration of infection, type of microorganism, whether or not the implant has loosened, and signs of osteitis [12].Treatment consists of antibiotic therapy (specifically targeting *Staphylococcus aureus*) combined with either early debridement and irrigation with replacement of mobile components, or complete removal of the implant, with or without subsequent reimplantation. While trapeziectomy remains a safe and reliable option in cases of infection requiring implant removal, revision surgery may be the best option for younger and more active patients [77].The major point in revision is to assess if there is concomitant loosening of the implant, as pointed out by Chammas et al. [78]. Secondly, it must be verified if the prosthesis is still available. In this latter case, if there is a concomitant destruction of the trapezium revision is not possible trapeziectomy must be performed, otherwise bipolar revision with a new TCM prosthesis can be pursued.

Despite recent advancements, such as standardized protocols for TMC prosthesis loosening [78], no consensus exists regarding infection management. This review highlights the need for dedicated studies to establish clear diagnostic and therapeutic protocols for TMC prosthetic infections. Future research should focus on stratifying infections by onset, identifying specific risk factors, and optimizing treatment strategies tailored to the unique challenges of TMC prostheses. With improved reporting and standardized approaches, surgeons can better address this rare but clinically significant complication.

### Study Limitations

This review is limited by the predominance of retrospective studies and small cohorts, reducing the generalizability of findings. Heterogeneity in diagnostic criteria, reporting of infections, and treatment strategies complicates comparisons and standardization. Many studies lacked detailed data on infection timing, pathogens, and long-term outcomes. Variability in implant designs, surgical techniques, and postoperative protocols further limits the consistency of results. Additionally, publication bias may underestimate the true incidence of complications.

Future research with standardized methodologies and larger multicenter trials is needed to address these gaps and enhance infection management in TMC prosthesis surgery.

## 5. Conclusions

The management of infections TMC prosthesis remains a complex and underexplored area within hand surgery. While the overall infection rate is relatively low compared to larger joint replacements, the lack of consensus on classification, diagnostic criteria, and treatment protocols poses significant challenges.

The heterogeneity of patient characteristics, implant designs, and clinical presentations underscores the necessity of a personalized and precision medicine approach in both prevention and management. Current evidence suggests that early identification and prompt, tailored intervention—whether medical, surgical, or a combination of both—are critical for preserving joint functionality and patient quality of life. Individualized treatment strategies, integrating patient-specific risk factors, microbiological data, and functional demands, may optimize outcomes and reduce unnecessary implant removal. Future research should focus on developing standardized guidelines for infection management and stratifying risk factors to improve outcomes. This review highlights the necessity of a multidisciplinary approach and underscores the importance of individualized care to address the nuanced complications of TMC joint prosthesis infections.

## Figures and Tables

**Table 1:** Inclusion and exclusion criteria.

	Inclusion	Exclusion
**Population**	Patients with trapeziometacarpal (TMC) joint prostheses	Studies focusing on other inflammatory conditions (e.g., tendinitis)
**Intervention**	Any surgical treatment for rhizarthrosis involving prostheses	Non-surgical interventions or unrelated procedures
**Design**	RCTs, clinical trials, case series with ≥10 patients, and case reports or case series with <10 patients if reporting PJI	Systematic reviews, meta-analyses, in vitro/animal/cadaver studies, technical notes, commentaries
**Other**	English, Spanish, French, Portuguese and Italian	Not in English, Spanish, French, Portuguese and Italian

**Table 2:** Demographic characteristics of the analyzed patients (NS: not specified).

Ref.	Year of Publication	Type of Studies	N° Patients	Age	N° Male	N° Female
Andrzejewski A [11]	2019	Retrospective	93	59.5	20	73
Bricout M [12]	2016	Retrospective	139	62.7	20	119
Chiche L [13]	2023	Retrospective	157	65	25	166
Degeorge B [9]	2018	Retrospective	41	66.3	3	38
Vanmierlo B [14]	2022	Retrospective	46	67.1	17	29
Tchurukdichian A [15]	2023	Retrospective	211	54.7	44	167
Gonzalez-Espino P [16]	2021	Retrospective	92	62.64	8	84
Krukhaug Y [17]	2014	Retrospective	82	62.5	29	53
Dreant N [18]	2019	Retrospective	25	63.4	2	23
Froschauer SM [19]	2020	Retrospective	29	54	3	26
Pritchett JW [20]	2012	Prospective	124	63	31	107
Buffet A [21]	2022	Retrospective	19	69	19	0
Fauquette PJ [22]	2023	Retrospective	66	59.3	6	60
Falkner F [23]	2023	Prospective	52	58	14	38
Toffoli A [24]	2024	Retrospective	76	67	10	66
Toffoli A [25]	2017	Retrospective	80	68	11	69
Vissers G [26]	2019	Prospective	24	71	2	22
Martins A [27]	2020	Retrospective	46	68	7	39
Frey PE [28]	2024	Retrospective	26	64	9	22
Lussiez B [29]	2021	Prospective	107	64.5	21	86
Tchurukdichian A [30]	2020	Prospective	95	61	13	82
Martin-Ferrero M [31]	2020	Prospective	199	59	10	188
Dehl M [32]	2017	Retrospective	84	71	6	78
Regnard PJ [33]	2006	Retrospective	100	59	15	85
Hansen TB [34]	2008	NS	9	58.3	3	7
Hansen TB [35]	2008	NS	16	54	1	15
Kollig E [36]	2017	Retrospective	26	63	NS	NS
Thorkildsen RD [37]	2019	Retrospective	20	64	6	14
Ten Brinke B [38]	2021	Prospective	10	72	1	9
Semere A [39]	2015	Prospective	51	58.2	3	48
Klim SM [40]	2023	Prospective	82	56	13	69
Klahn A [41]	2012	Prospective	37	56.5	5	32
Tchurukdichian A [42]	2021	Prospective	179	66	37	142
Froschauer SM [43]	2020	Retrospective	29	54.4	5	24
Pérez-Ubeda MJ [44]	2003	Prospective	19	65	2	17
Lemoine S [45]	2009	Retrospective	68	61	13	55
Pendse A [46]	2009	Retrospective	50	64.5	14	36
Ulrich-Vinther M [47]	2008	Prospective	42	58	NS	NS
Martin-Ferrero M [48]	2014	Prospective	60	58	3	57
MacDermid JC [6]	2003	Retrospective	25	64	4	21
Kaszap B [49]	2012	Retrospective	12	64	3	9
Hariri A [50]	2011	Retrospective	1	58	0	1
Robles-Molina MJ [51]	2017	Retrospective	31	56.37	4	27
Barrera-Ochoa S [52]	2017	Case report	1	69	0	1
Rein S [53]	2024	Case report	1	58	0	1
Dumartinet-Gibaud R [54]	2020	Retrospective	63	59	8	55
Hernández-Cortés P [55]	2012	Retrospective	19	57	0	19
Goubau JF [5]	2013	Prospective	22	66	1	21
De Smet A [56]	2020	Prospective	57	56	12	61
Badia A [57]	2006	Prospective	25	71	1	24
Thillemann JK [58]	2016	Retrospective	40	58	8	32
Schmidt I [59]	2014	Case report	1	64	0	1
Vandenberghe L [60]	2013	Retrospective	90	56	4	86
Gómez-Garrido D [61]	2019	Retrospective	137	61.6	35	102
Cootjans K [62]	2017	Prospective	156	58	15	101
Thorkildsen RD [63]	2020	Retrospective	5	62	0	5
Van Royen K [64]	2021	Case report	1	57	0	1
Duché R [65]	2022	Retrospective	NS	/	NS	NS
de Jong TR [66]	2023	Retrospective	29	59	0	29
Cebrian-Gomez R [8]	2019	Prospective	84	60.4	7	77
Caekebeke P [67]	2018	Retrospective	35	57	16	19
Johnston P [68]	2012	Retrospective	26	53.3	NS	NS

**Table 3:** Infection rate and treatments (NS: not specified).

Ref.	Type of Prosthesis	N° Infected Patients	SSI	Deep	Isolated Pathogen	Resolution with Only Antibiotic Therapy	Surgical Treatment	Type of Surgery
Andrzejewski A [11]	Maia	2	0	2	NS	No	2	Trapeziectomy
Bricout M [12]	Maia	2	0	2	*S. aureus*	No	2	NS
Gonzalez-Espino P [16]	Touch	1	1	0	NS	Yes	No	/
Pritchett JW [20]	Bio Pro Modular Thumb Implant	1	0	1	NS	/	Yes	NS
Thorkildsen RD [37]	Elektra	1	0	Suspected	NS	No	Yes	Debridement + bone cement spacer; arthrodesis 6 months later
Klahn A [41]	Elektra	1	1	0	NS	Yes	0	/
Froschauer SM [19]	Ivory	1	0	1	NS	No	Yes	Prosthesis explantation and surgical conversion into a resection arthroplasty
Pendse A [46]	Guepar	2	2	0	NS	/	/	/
MacDermid JC [6]	Swanson	1	0	1	*S. aureus*	/	Yes	Removal
Thillemann JK [58]	Motec	1	0	1	NS	No	Yes	Trapeziectomy
de Jong TR [66]	Maia	1	1	0	/	/	/	/
Cebrian-Gomez R [8]	Ivory	1	1	0	/	/	/	/

## Data Availability

The original contributions presented in this study are included in the article/Supplementary Materials. Further inquiries can be directed to the corresponding author.

## Supplementary Materials

The following supporting information can be downloaded at: https://www.mdpi.com/article/10.3390/jpm16010035/s1, Table S1: PRISMA 2020 Checklist.

## Abbreviations

TMC	Trapeziometacarpal
CMC	Carpometacarpal
LRTI	Ligament Reconstruction and Tendon Interposition
RCT	Randomized Controlled Trial
SSI	Surgical Site Infection
CDC	Centers for Disease Control and Prevention
CRP	C-Reactive Protein
ESR	Erythrocyte Sedimentation Rate
WBC	White Blood Cell count
PRISMA	Preferred Reporting Items for Systematic Reviews and Meta-Analyses
PROSPERO	International Prospective Register of Systematic Reviews
SPSS	Statistical Package for the Social Sciences
